# Adsorption and selectivity studies of direct and magnetite-cored molecularly imprinted polymers (MIPs and magMIPs) towards chosen chalcones investigated with various analytical methods

**DOI:** 10.1039/d1ra03391c

**Published:** 2021-07-21

**Authors:** Mateusz Pawlaczyk, Maria Guć, Grzegorz Schroeder

**Affiliations:** Faculty of Chemistry, Adam Mickiewicz University in Poznań Uniwersytetu Poznańskiego 8 61-614 Poznań Poland mateusz.pawlaczyk@amu.edu.pl +48 61 829 17 97

## Abstract

The following article presents a method for obtaining molecularly imprinted polymers (MIPs) dedicated to *trans*-chalcone (TC) and 2′,4′-dihydroxy-3-methoxychalcone (DHMC). The synthetic protocol optimized with a choice of the most suitable functional monomer led to the synthesis of MIPs and their non-imprinted equivalents (NIP) performed *via* direct polymerization or on the surface of magnetite nanoparticles. The characterized materials were investigated for adsorption isotherms of TC and DHMC, which led to satisfactory values of maximal adsorption capacity, reaching 131.58 and 474.71 mg g^−1^, respectively. Moreover, all the polymers were studied for the adsorption kinetics and the selectivity towards four structurally different chalcones, which proved the proper selectiveness towards the template molecules. Also, the kinetic profiles of chalcones' adsorption on the synthesized MIPs showed a quasi-plateau reached already after 2 hours, indicating high sorption effectiveness. The studies involved the use of various analytical techniques, which afforded a comprehensive and reliable description of the materials' adsorption efficacy. It was found that the materials successfully bind the MIP-complementary analytes and also structurally similar chalcones, with slightly lower intensity.

## Abstract 2

The following article presents a method for obtaining molecularly imprinted polymers (MIPs) and their magnetic equivalents (magMIPs) using chalcones as imprinting molecules, and their application for direct and competitive chalcones' adsorption.

## Graphical abstract

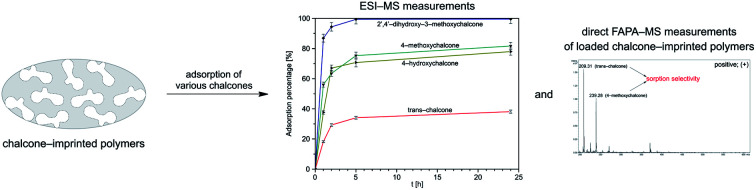

## 1. Introduction

Discovering and designing of biologically active molecules exhibiting multitherapeutic effects is still of great interest. Among a plethora of molecules proved to be efficient agents in various diseases' treatment, chalcones are very attractive candidates for clinical applications. The term “chalcone” was proposed in 1899 by Kostanecki and Tambor and referred to a molecule built of two phenyl rings connected by a three-carbonyl enone chain.^1^ Further exploration of natural and synthetic molecules based on the structure of benzylidene acetophenone (chalcone) has led to a broadening of the term, now indicating the pure molecule and its multiple derivatives.

Although chalcones have been found to naturally occur in many species of plants, vegetables, and fruits, the greatest amounts of chalcone and its derivatives have been isolated from families of Leguminosae (peas and beans), Asteraceae (*e.g.* asters, daisies, or sunflowers), and Moraceae (*e.g.* mulberry, jackfruit, or fig).^2^ Up to now, chalcones have been postulated for multiple therapeutic effects, especially anticancer, anti-inflammatory, antioxidant, antitumor, anti-bacterial, anti-viral, and anti-fungal activities.^3–8^ Moreover, several chalcone-derivatives have been investigated and proved for efficient inhibition of key enzymes responsible for viral replication of SARS-CoV, HIV, HBV, or HCV.^9^

The bioactivity of chalcones is undoubtedly based on their structural features, especially the position and the type of substituents in both phenyl rings, such as saturated or unsaturated hydrocarbon groups, halogens atoms, additional aromatic or heteroaromatic rings, bulky cyclopropyl or adamantane domains, as well as hydroxyl, carboxylic or amide groups.^10,11^ It is worthy to highlight that incorporation of functional groups into phenyl rings, which affords a planarity of the final molecule, provides an intrinsic fluorescence, making chalcones applicable for easy visualization of targeted tissues, cells, or bioconstituents.^12^

Even though chalcones, as naturally occurring species, present remarkably attractive bioactivity, the development of new methods for their extraction or selective absorption, and determination of bioactive chalcones in various complex matrices, is still an important scientific problem, since the separation technique must be coupled with a developed analytical method, enabling a qualitative and quantitative determination of analytes in each stage.^13,14^ The use of a particular sorptive material for the extraction of chalcones from various matrices is mostly determined by acceptable purification performance and high extraction efficiency. Goncalves *et al.* investigated the efficiency of five different versatile non-specific packed sorbents towards various analytes, including chalcones.^15,16^ The studies showed that silica sorbents functionalized with aliphatic chains (C_2_, C_8_, and C_18_) are suitable for extraction of non-polar analytes, while the studied polymer resin or silica containing cationic-exchange domain are efficient towards polar analytes. The non-specific sorbents showed moderate chalcones' adsorption performance depending on their structures and types of pendant functional groups in the chalcone skeleton.

The reliable analytical methods demand both selective and sensitive recognition of the target analytes, and instrumental methods analysis of biocomponents. These requirements can be met using a combination of molecularly imprinted polymers (MIP) as analytes' binding matrices, and mass spectrometry. Molecular imprinting is a process of formation of cavities in the polymer structure, which can be understood as selective recognition sites that are complementary to a chosen template/analyte molecule by size, shape, and pendant functional groups. Therefore, the selectivity of the designed MIP towards the particular analyte is achievable through a “lock-and-key” mechanism.^17–19^ The process of the polymer molecular imprinting involves a crucial step of formation of a complex between a template and a functional monomer, usually through non-covalent interactions (*e.g.* dipole–dipole interactions, hydrogen bonding, or hydrophobic effects) in a pre-polymerized mixture. As obtained rigid polymeric matrices contain cavities complementary to the used template molecules.^20–24^ The conducting of the described polymerization process in the presence of nanoparticles, in particular of superparamagnetic iron oxide nanoparticles (Fe_3_O_4_; SPIONs), allows for the obtaining of polymeric hybrid materials exhibiting beneficial magnetic susceptibility.^25,26^

Herein, we present the method of obtaining the polymers imprinted with *trans*-chalcone and 2′,4′-dihydroxy-3-methoxychalcone, as two the most structurally different among all the chalcones used during studies. In order to optimize and achieve the highest chalcone-imprinting efficiency, three standard monomers (AA, MAA, and 4-VP) were used for preliminary tests of adsorption properties. Bare molecularly-imprinted polymers and their magnetic equivalents, as well as non-imprinted polymers, were further investigated for their adsorption rate and efficiency using several analytical techniques, including UV-vis spectroscopy and mass spectrometry working in two different ionization methods, *i.e.* electrospray (ESI) and flowing atmosphere-pressure afterglow (FAPA) ionizations. The use of various analytical techniques allowed to perform unambiguous validation of chalcones determination processes using the synthesized selective polymeric sorbents.

## 2. Materials and methods

### 2.1. Chemicals

The chalcones used (*trans*-chalcone (TC), 97%; 4-methoxychalcone (MC), 98%; 2′4′-dihydroxy-3-methoxychalcone (DHMC), 97%; and 4-hydroxychalcone (HC), 97%) were obtained from Alfa Aesar (Haverhill, MA, USA). The functional monomers – acrylamide (AA; ≥ 98%), 4-vinylpyridine (4-VP; 95%, containing 90–110 ppm MEHQ as inhibitor), and methacrylic acid (MAA, 99%, containing 250 ppm MEHQ as inhibitor); the polymerization initiator – 2,2′-azobis(2-methylpropionitrile) (AIBN, 98%); the cross-linking agent – ethylene glycol dimethacrylate (EGDMA, 98%, containing 90–110 ppm MEHQ as inhibitor); tetraethyl orthosilicate (TEOS; ≥ 99%); (3-trimethoxysilyl)propyl methacrylate (MPS; ≥ 97%); and *N*-ethyldiisoproylamine (DIPEA ≥ 98%) were purchased from Sigma-Aldrich (Saint Louise, MO, USA). The reagents containing MEHQ impurities as inhibitors were purified before their use using basic activated Al_2_O_3_ Brockamnn also obtained from Sigma-Aldrich. Iron(ii) precursor (NH_4_)_2_Fe(SO_4_)_2_·6H_2_O of purity grade p.a. was purchased from Aktyn (Suchy Las, Poland). Iron(iii) precursor FeCl_3_·6H_2_O (≥97%); PBS buffer ingredients Na_2_HPO_4_·2H_2_O (>99%), NaH_2_PO_4_·H_2_O (>99%), and KCl (99.5%), and the solvents of purity grade p.a. (CHCl_3_ ≥ 98.5%; anhydrous EtOH 99.8%; and NH_4_OH 25%) were supplied by POCH (Gliwice, Poland), while AcOH 80%, DCM ≥ 99%, MeOH ≥ 99.8%, NaCl, and buffer solutions of pH 2.0, 5.0, and 10.0 were obtained from Eurochem (Tarnów, Poland).

### 2.2. Instruments

All the molecularly imprinted and non-imprinted polymers were characterized with FT-IR analysis, analyzing polymers' suspension in KBr pellets in Bruker IFS 66v/S (Bremen, Germany) spectroscope. The spectra were recorded in the range of 400–4000 cm^−1^. Thermal stability of the materials was investigated by thermogravimetric measurements conducted in Setaram Setsys 1200 analyzer (Caluire, France), which operated in the temperature range between 20 and 1000 °C. The samples were heated with a heating rate of 5 °C min^−1^. In order to investigate Fe_3_O_4_-based materials' composition and size, they were subjected to XRD analysis using Bruker D8 Advance (Bremen, Germany) apparatus. The diffractometer was equipped with Cu K_α1_ X-ray energy source (*λ* = 1.5406 Å) and operated in 2*θ* range of 6–60° as a high-angle mode. Also, the chosen materials were characterized with BET analysis performed using Quantachrome Autosorb iQ (Boynton Beach, USA) analyzer. The samples of 150 mg were outgassed for 12 hours at 100 °C prior to the final N_2_ adsorption/desorption analysis conducted for 75 hours at a temperature of 77.35 K.

The isothermal studies of the chosen chalcones adsorption on the synthesized polymers were monitored by UV-vis measurements, performed using Agilent 8453 spectrophotometer (Santa Clara, USA). The spectra of solutes after polymers' treatment were recorded in the wavelength range between 250 and 1000 nm, with a wavelength resolution of 1 nm. The cuvettes of standard thickness (optical path length of 1 cm) used for spectrophotometric measurements were made of PMMA in order to avoid any spectral disturbances. Each spectrum was made in triplicate, taking the mean value of absorbances as an analytical signal. The kinetics of chalcones' adsorption on the polymers was monitored using amaZon SL ion trap (Bruker, Bremen, Germany) mass spectrometer equipped with electrospray ionization source operating in infusion mode (ESI-MS). The spectrometer utilized helium and nitrogen as the cone and the desolvating gases, respectively, while the source temperature was set at 80 °C and the desolvation temperature at 250 °C. The apparatus worked at the conditions of −4.5 kV capillary voltage and −0.5 kV endplate offset. The solutions of chalcones remaining after the adsorption processes were injected into the ionization source using a syringe pump, with a flow rate set at 10 μL min^−1^. The spectra were recorded in the mass range between 100 and 1000 *m*/*z*, in both positive and negative ionization modes. Moreover, the complexes of polymers and chalcones were subjected to direct analysis using L-FAPA ambient plasma ion source (ERTEC, Wroclaw, Poland). The ionization source was placed and operated at a distance of ∼10 cm from the spectrometer inlet. The generated external plasma and elevated temperature, afforded by crucible placed approximately 1 cm below the plasma source, led to the ionization of the adsorbed chalcones' molecules, and their direct infusion to the spectrometer's analyzer. The crucible allowed the controlled temperature increase of the materials in the range of 20–300 °C. The analysis conditions remained the same as described for ESI-MS analysis.

### 2.3. Synthesis of molecularly imprinted polymers and their magnetic analogues

#### 2.3.1. Synthesis of MIPs and NIPs containing different monomers

The general procedure for the synthesis of *trans*-chalcone imprinted polymers containing different monomers was as follows: 1 mmol of a template (*trans*-chalcone (TC) – 208.2 mg), 4 mmol of a chosen monomer (acrylamide, AA – 284.3 mg; methacrylic acid, MAA – 337.5 μL; or 4-vinylpyridine, 4-VP – 425.7 μL), and 20 mmol of a cross-linking agent (ethylene glycol dimethacrylate, EGDMA – 3.776 mL) were dissolved in 25 mL of anhydrous chloroform in a glass bottle. The mixture was sonicated for 30 minutes at room temperature with a constant purging inflow of nitrogen. Then, an initiator (azobisisobutyronitrile, AIBN – 1 mL) was added, and sonication under anhydrous conditions was maintained for the next 20 minutes. Afterwards, the bottle was closed fast and tight with a cap, not to let oxygen diffusion to the mixture, and placed in an oven for 24 hours at 60 °C. The obtained polymer was dried under vacuum at 50 °C and ground in an agate mortar. The same procedure was adopted for the synthesis of NIPs, except for the template's addition step, yielding in white powders of (AA)–, (MAA)–, and (4-VP)–TC–MIP and complementary monomer-differing NIPs. Furthermore, for the template's elution, the obtained MIPs were subjected to Soxhlet extraction for 5 days, using ∼250 mL of EtOH : AcOH (25 : 1) solution. The eluent was replaced every 24 hours.

#### 2.3.2. Synthesis of acrylamide-based MIPs

The procedure for the synthesis and the treatment of MIPs using AA as the monomer, and *trans*-chalcone or 2′,4′-dihydroxy-3-methoxychalcone as templates was used as described in paragraph 2.3.1, with certain amounts of reagents: AA – 284.3 mg, *trans*-chalcone (TC) – 208.2 mg, and 2′,4′-dihydroxy-3-methoxychalcone (DHMC) – 270.3 mg. The synthetic steps and the amounts of the other reagents stayed unaltered, obtaining white solids of TC–MIP and NIP, and a yellow solid of DHMC–MIP.

#### 2.3.3. Synthesis of Fe_3_O_4_–MPS

The synthesis of Fe_3_O_4_ magnetic nanoparticles and their encapsulation with silica matrix was conducted according to the procedure described before.^27^ Briefly, to an aqueous mixture of FeCl_3_·6H_2_O (30 mmol; 8.11 g) and (NH_4_)_2_Fe(SO_4_)_2_·6H_2_O (15 mmol; 5.88 g) purged with nitrogen at room temperature, an aqueous solution of ammonia was added dropwise, immediately forming a black, magnetically responsive precipitate. The obtained Fe_3_O_4_ nanoparticles were washed several times with deionized water and methanol and then separated and dried under vacuum, with a yield of 99%. The encapsulation was performed by addition of 1.5 mmol of TEOS (332.4 μL) to a suspension of 3 g of the obtained Fe_3_O_4_ nanoparticles in H_2_O : EtOH (2 : 1) mixture with 10 mL of ammonia at 45 °C. The mixture was sonicated for 3 hours at elevated temperature and then mixed at room temperature overnight. The solid wash separated, washed with H_2_O : EtOH (2 : 1) solution and EtOH several times, and subsequently dried under vacuum. Afterwards, the obtained Fe_3_O_4_–TEOS particles were sonicated in anhydrous toluene at 65 °C for 1 hour. Then, 5 mL of DIPEA was added, and a solution of MPS (6 mmol; 1.45 mL) in 25 mL of toluene was added dropwise. The mixture was further sonicated at elevated temperature for 6 hours, and then shaken at room temperature for 16 hours. The dark brown particles were separated, washed with toluene and DCM several times, and dried under vacuum, yielding in Fe_3_O_4_–MPS.

#### 2.3.4. Synthesis of acrylamide-based magMIPs

The preparation of MIPs with magnetic Fe_3_O_4_ core (magMIPs) was performed by sonication of a mixture of 200 mg of Fe_3_O_4_–MPS, 1 mmol of the template (TC – 208.2 mg, or DHMC – 270.3 mg), 4 mmol of the monomer (AA – 284.3 mg) and 20 mmol of the cross-linking agent (EGDMA – 3.776 mL) dissolved in 30 mL of anhydrous chloroform in a glass bottle for 30 minutes at room temperature, with a constant flow of N_2_ through the mixture. Afterwards, the initiator (AIBN – 1 mL) was added and the temperature of the ultrasound bath was elevated to 60 °C. The mixture was sonicated in the given conditions for 1 hour. Then, the bottle was sealed with a cap and placed in the oven at 60 °C. The content was regularly shaken to avoid sedimentation of magnetite particles, until the full gelation. The polymerization process was handled for 24 hours, with subsequent drying under vacuum and grinding in the agate mortar, obtaining brown powders of TC–magMIP, DHMC–magMIP, and magNIP.

### 2.4. Evaluation of imprinting factors (IF) of *trans*-chalcone-imprinted MIPs with different monomers

The polymers obtained as described in paragraph 2.3.1 were subjected to investigation of their imprinting factor, which was performed as follows: 10 mg samples of (AA)–, (MAA)–, and (4-VP)–TC–MIP and complementary NIPs were mixed with 10 mL of 0.1 mM *trans*-chalcone solution in EtOH : H_2_O mixture (1 : 1) for 24 hours at room temperature. After that time, the polymers were centrifuged and the solutes were investigated for the amount of remaining TC, using UV-vis measurements with a detection maximum at 307 nm. The concentration of unbound TC was determined from a pre-performed calibration curve. Substraction of this value from the initial analyte concentration gave the amount of analyte bound *B* in the sample mass [mg g^−1^]. To investigate non-specific surface binding, static binding assays were further conducted on both the MIPs and NIPs in parallel.
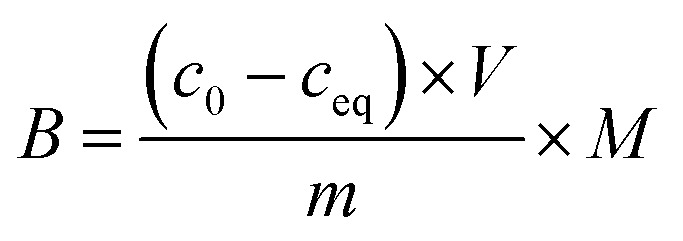


All the calculated *B* values were further used for investigation of imprinting factors (IF) values and selectivity (*α*) of MIPs containing different monomers, which were derived from the below equations:
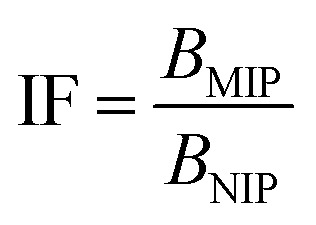

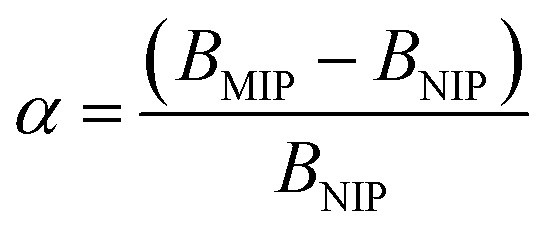
where *B*_MIP_ and *B*_NIP_ relate to *B* values calculated for MIP containing particular monomer (MAA, AA, or 4-VP) and the corresponding NIP, respectively.

### 2.5. Investigation of pH influence on adsorption processes

In order to evaluate an influence of solution pH on the polymers' adsorptive properties towards dihydroxy-derivative of chalcone, 15 mg samples of polymers obtained as described in paragraphs 2.3.2. and 2.3.4. were incubated in 10 mL of 0.1 mM solution of DHMC prepared of 1 mL of 1 mM DHMC solution in EtOH : H_2_O mixture (1 : 1) and 9 mL of different buffers of pH 2.0, 5.0, 7.4, or 10.0. The materials were incubated in the chalcone's solutions for 24 hours, and then separated by centrifugation and/or using a magnet. Concentrations of chalcone remaining in the solutions were quantified using ESI-MS analysis.

### 2.6. Adsorption isotherms of acrylamide-based MIPs towards TC and DHMC

The adsorption isothermal studies were based on the interaction between a certain amount of each of MIPs, magMIPs, and NIPs with solutions of analytes of different concentrations. Namely, 10 mg samples of each material were poured to 10 mL of *trans*-chalcone (TC) or 2′,4′-dihydroxy-3-methoxychalcone (DHMC) solutions in mixture EtOH : H_2_O (1 : 1) of concentrations as follows: 0.01; 0.025; 0.05; 0.1; 0.5; and 1 mM. The mixtures were incubated at room temperature for 24 hours. Afterwards, the solids were centrifuged, and the filtrates were subjected to UV-vis analysis. The final concentrations of the chalcones remaining in the solutions (*c*_eq_) were calculated on the basis of the absorption maxima values at *λ* = 307 and 383 nm assigned for TC and DHMC, respectively. The calculated *c*_eq_ values [mM] were used for the calculation of *q*_eq_, which is the amount of the chalcone bound to the material [mg g^−1^], using the below equation, where *m* is the sample mass [mg], *V* is the volume of the chalcone solution used [mL], and *M* is the molar mass of the particular chalcone [g mol^−1^]:
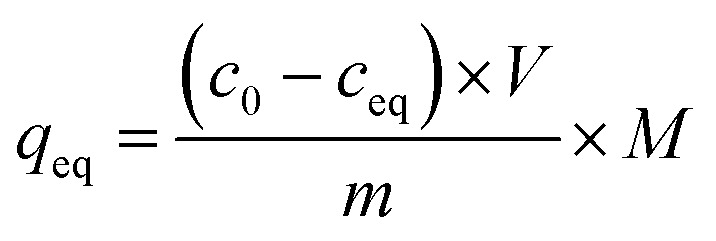


In order to investigate the adsorptive properties of the synthesized NIPs, MIPs, and their magnetic analogues towards the template molecule or structurally different analyte, the obtained isothermal data were fitted to the Langmuir and the Freundlich isotherms model, which linear plots are presented below in the proper manner:
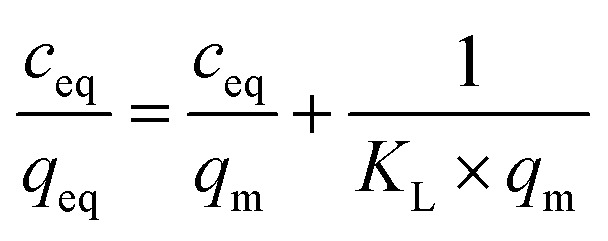

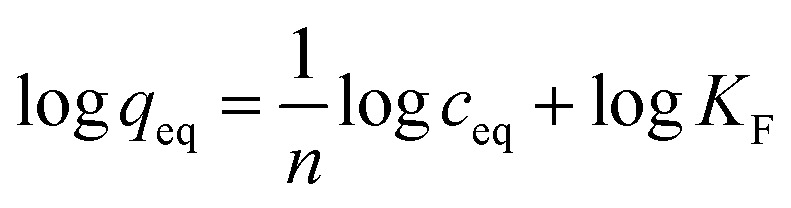
where *q*_m_ is the maximal adsorption capacity of the material towards the particular analyte [mg g^−1^], *K*_L_ is the Langmuir constant dependent on the adsorption energy [L mg^−1^], 1/*n* is the empirical constant connected with the adsorption intensity [−], and *K*_F_ is the Freundlich constant [mg g^−1^ (L mg^−1^)^1/*n*^].

Moreover, the obtained complexes of polymers with trapped template molecules (15 mg) were incubated in 5 mL of four different buffer solutions of pH 2.0, 5.0, 7.4, and 10.0 for 2 hours at room temperature. After the incubation time, the solids were separated and the solutes were subjected to UV-vis analysis, in order to establish the amount of the chalcones released to the desorbing media.

### 2.7. Selectivity of acrylamide-based MIPs and magMIPs towards various chalcones

Investigation of the selectivity of the synthesized polymers towards four chalcones: *trans*-chalcone (TC), 2′,4′-dihydroxy-3-methoxychalcone (DHMC), 4-hydroxychalcone (HC), and 4-methoxychalcone (MC) was performed by addition of 10 mL of a mixture of all the chalcones in EtOH : H_2_O (1 : 1) solution at their final concentration of 0.1 mM to 10, 20, or 30 mg samples of the polymers. The solution-sample mixtures were shaken for 24 hours at room temperature. Subsequently, the materials were centrifuged, and the filtrates were subjected to ESI-MS analysis in both, positive and negative, modes. The final adsorption percentage values for each of the chalcones were calculated on the basis of pre-performed calibration curves for ESI-MS analysis. Moreover, the dried polymer–chalcones samples of 5 mg were also subjected to FAPA-MS analysis ran in positive and negative modes.

For the mixtures containing 10 mg samples of the materials, 50 μL aliquots of solutes were collected after 1, 2, 5, and 24 hours of incubation injected to the mass spectrometer, and the concentration of the chalcones remaining in solution was calculated, in order to investigate the kinetics of chalcones' adsorption.

## 3. Results and discussion

### 3.1. Choice of the most efficient functional monomer

The synthetic protocol for obtaining the designed chalcone-imprinted MIPs and their magnetic equivalents was based on the standard two-step process involving non-covalent imprinting, followed by a formation of a polymeric net. The imprinting efficacy is strongly dependent on non-covalent interactions, which stabilize complexes formed between functional groups of the monomers used and functional groups of analyte – herein, *trans*-chalcone. The chosen monomers, which were acrylamide, methacrylic acid, and 4-vinylpyridine, containing electrodonating amine group, carboxyl group, and pyridyl nitrogen atom, respectively, considerably differ in the ability to interact with a free carbonyl group of *trans*-chalcone. Therefore, an influence of the monomers used on the *trans*-chalcone imprinting efficiency, and thus *trans*-chalcone binding ability, was investigated. Table 1 presents the binding parameters of the synthesized and purified MIPs containing three different monomers (AA, MAA, and 4-VP), and their non-imprinted equivalents. *B*_MIP_ and *B*_NIP_ values correspond to the amount of TC adsorbed on the particular MIPs and NIPs, respectively. Moreover, the ratio of the adsorption capacities of MIPs containing particular monomers to the capacities of corresponding NIPs is called imprinting factor (IF) and indicates the effectiveness of the monomers in the molecular imprinting process. IF values collected in Table 1 are 1.10 for MAA, 4.75 for AA, and 1.90 for 4-VP, which indicates the highest binding efficiency of the polymers containing acrylamide as the monomer. Such phenomenon refers to the presence of a free amine group, which readily forms hydrogen bonding with free carbonyl groups of chalcones. Schwarz *et al.* performed modeling calculations of predicted theoretical energy of monomer-template complexes formation, between *trans*-resveratrol (a structurally similar molecule to *trans*-chalcone in shape and size) and various functional monomers.^28^ For the calculations involving the use of 4 equivalents of monomer, which was adopted during the current study, the highest complex stability was ascribed for acrylamide-complex, which is consistent with the calculated IF values. Moreover, the binding selectivity value *α* calculated for AA is several times higher than those calculated for MAA or 4-VP, proving its highest efficiency. Therefore, acrylamide was chosen as the most suitable monomer for the synthesis of chalcone-imprinted polymers.

**Table 1:** Binding parameters of the synthesized MIPs containing various monomers towards template molecule (*trans*-chalcone)

Monomer	*B*_MIP_ [mg g^−1^]	*B*_NIP_ [mg g^−1^]	IF	*α*
MAA	74.97	68.31	1.10	0.10
AA	130.58	27.49	4.75	3.75
4-VP	69.98	36.86	1.90	0.90

### 3.2. Synthesis and characterization of acrylamide-based MIPs

**Fig. 1:**
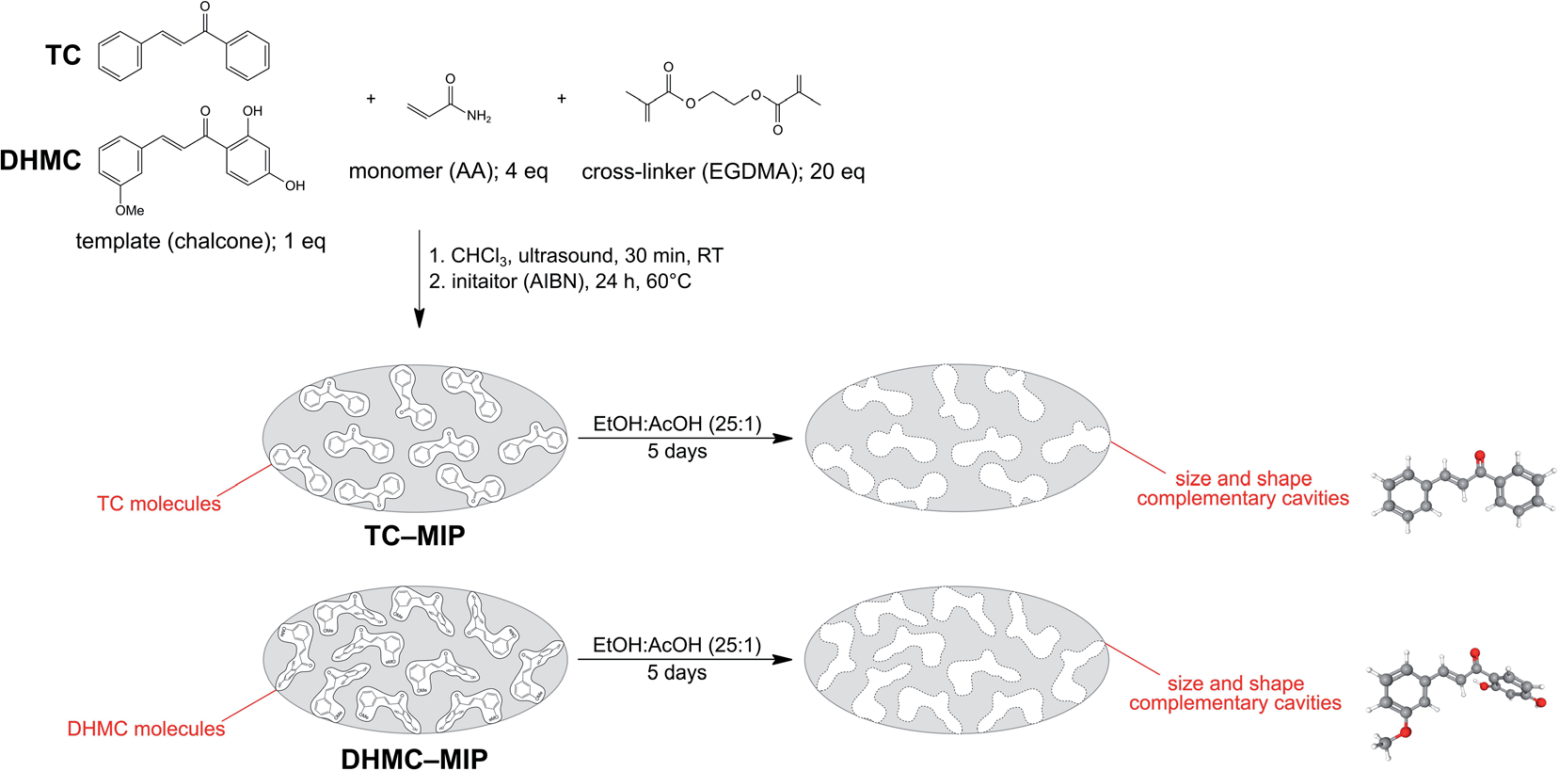
The scheme of molecularly-imprinted polymers synthesis and template removal, presented on the examples of *trans*-chalcone- and 2′,4′-dihydroxy-3-methoxychalcone-imprinting.

The synthetic procedure for obtaining molecularly-imprinted polymers is widely known, and is based on three crucial steps: (1) formation of a complex between the functional monomer and an imprinted template molecule through non-covalent interactions; (2) thermal- or photo-induced polymerization; (3) template removal leading to empty cavities in the polymeric matrix, which size and shape are analogous to the shape of imprinting molecule. The current studies involved the synthesis of a series of MIPs and magMIPs dedicated to two chalcone molecules, using acrylamide as a monomer, EGDMA as a cross-linking agent, and AIBN as a thermal-polymerization initiator. Fig. 1 presents the schematic representation of TC– and DHMC–MIP syntheses, which clearly indicates the differences between the size and shape of complementary cavities. The most influential factor is the presence of hydroxyl groups in 2′ and 4′ positions of HMDC, which triggers steric hindrance, leading to the loss of the molecule coplanarity.^29^ The differences between the shape of the internal cavities and the structure of the analytes would also lead to weaker sorption potential of *trans*-chalcone-imprinted polymer's towards chalcone's *cis*-isomer, taking the structural differences and molecular volumes into account. The only difference for the synthesis of magMIPs was based on the addition of an exact amount of Fe_3_O_4_/SiO_2_–MPS at the very first synthesis step, and the longer sonication at elevated temperature in order to afford full arrangement of polymer constituents around the nanoparticles.

**Fig. 2:**
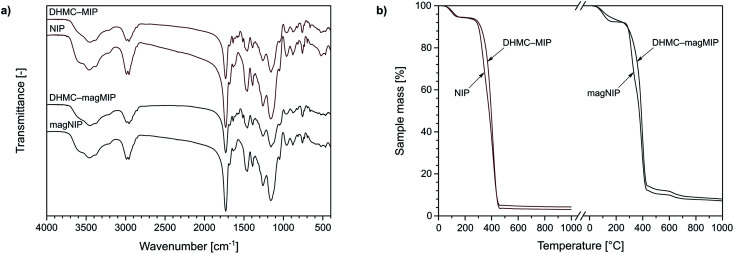
The characterization of DHMC–MIP and NIP, and their magnetic equivalents using: (a) FT-IR spectroscopy, and (b) thermogravimetric assays.

The characterization of the obtained materials was performed for exemplary MIPs, since the polymers were imprinted with *trans*-chalcone and its dihydromethoxy-derivative, therefore they differ only in insignificant extent. Nevertheless, the chosen materials have been characterized using various analytical techniques. For instance, TC–MIP and NIP, and their magnetic equivalents, were subjected to FT-IR measurements. All the spectra presented in Fig. 2a show several common signals, which correspond to the vibration of domains of the functional monomer or the cross-linking agent. The bands at 1159, 1260, 1390, and 1731 cm^−1^ are strictly related to C–C(O)–O stretching, C–O stretching, C–H bending of pendant CH_3_ groups, and C=O stretching of EGDMA domain as the cross-linker, respectively, while the signal at 1048, 1457, and 1684 cm^−1^ correspond to C–N stretching, N–H bending and C=O stretching of acrylamide residues as the monomer used, respectively. Moreover, the spectra present the signal at 759 cm^−1^, and the doublet at 2958 and 2987 cm^−1^ corresponding to C–H bending and stretching of multiple methylene and methyl groups, respectively, as well as broad bands centered at 3450 cm^−1^, which are connected with N–H stretching of hydrogen-bonded amine groups of acrylamide, as well as overtones of C=O stretching. The successful polymer imprinting with chalcone is proved by the appearance of new signals on the spectra of DHMC–MIP and DHMC–magMIP, which are: the stretching of C–H of aromatic rings at 661 cm^−1^, the stretching of C=C of chalcone phenyl rings at 1516 cm^−1^, and the stretching of conjugated C=O at 1637 cm^−1^. Also, each spectrum shows insignificant signals at 880 and 961 cm^−1^ related to C=C bending of trace amounts of unreacted EGDMA and acrylamide, respectively. The same polymeric representatives were characterized with thermogravimetric analysis, which curved are presented in Fig. 2b. Each of the TG curves shows the first decomposition step between 50 and 130 °C with a sample mass decrease of 5%, which is related to the loss of solvent traces and volatile reagents, such as the used initiator AIBN. The drastic mass loss of approximately 80–90% is observed in the region of 280 and 460 °C, which is connected to the slow oxidation of polymers' organic domains. For the polymers imprinted with 2′,4′-dihydroxy-3-methoxychalcone, within the given temperature range, a slight bulge is visible, indicating the oxidation of additional organic residues – the template molecules. For the magnetite-based polymers DHMC–magMIP and magNIP the final sample mass remains at the level of 10–15%, which is related to the use of thermally stable Fe_3_O_4_/SiO_2_ nanoparticles as the polymer's supports.

**Fig. 3:**
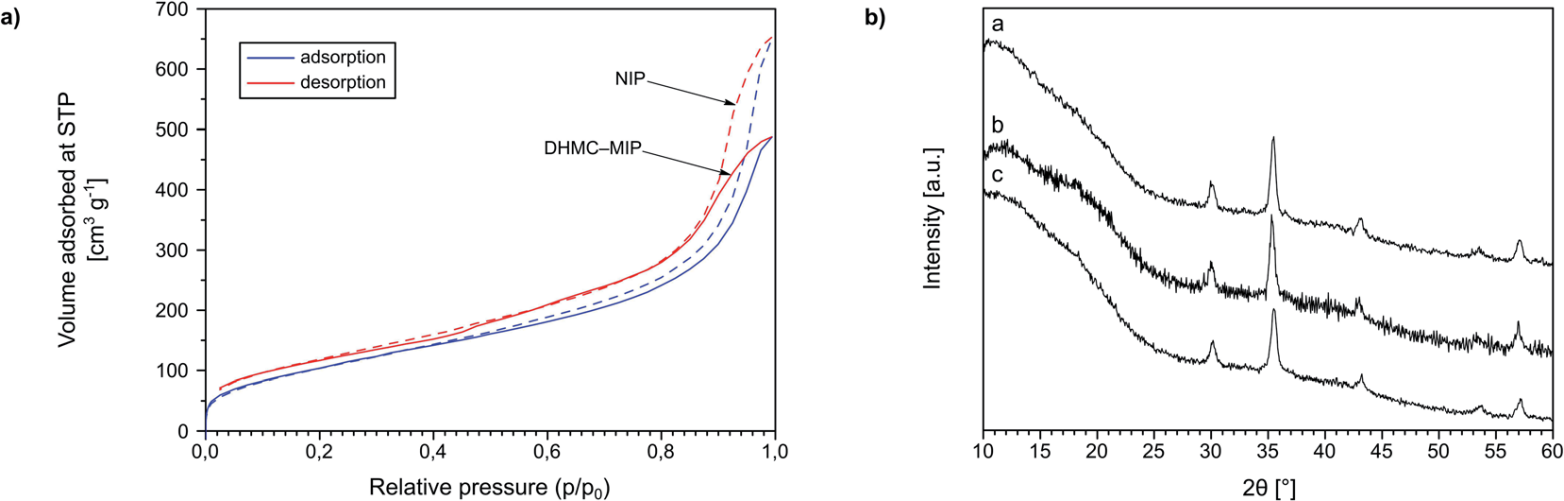
(a) the BET isotherms obtained for the polymer imprinted with 2′,4′-dihydroxy-3-methoxychalcone (DHMC–MIP), and for the non-imprinted polymer (NIP); (b) the XRD spectra of the synthesized magnetite-core polymers: (a) TC–magMIP, (b) DHMC–magMIP, and (c) magNIP.

Moreover, in order to show the imprinting efficiency, and thus the creation of molecular cavities within the polymer matrix, exemplary materials DHMC–MIP and NIP were subjected to nitrogen adsorption–desorption analysis (Fig. 3a). This widely used technique allows for assessing the porous properties of the samples, based on the BET model assuming a gas condensation in a form of a monolayer on the surface of materials, which amount is strictly correlated with the total surface area of accessible pores and cavities. One of the methods for creating of micro-sized pores is molecular imprinting, which is proved by the calculated micropore volume and area of 0.003 cm^3^ g^−1^ and 19.165 m^2^ g^−1^, respectively, for the polymer imprinted with 2′,4′-dihydroxy-3-methoxychalcone, while the non-imprinted polymer exhibits no microporosity. Moreover, N_2_ adsorption/desorption measurements led for the calculation of external surface area (for the BET model in the range of *p*/*p*_0_ between 0.1 and 0.3) which was 435.64 m^2^ g^−1^ for DHMC–MIP and 416.57 m^2^ g^−1^ for NIP, indicating the increase of the imprinted polymer surface area by incorporation of template micropores into the polymeric net. Additionally, the BJH method, which is applicable for gas adsorption assays, was implemented in order to investigate the volume of polymers' pores. Material DHMC–MIP was described with a mean pore volume of 0.670 cm^3^ g^−1^, while NIP with a value of 0.928 cm^3^ g^−1^, which proves the formation of smaller cavities within the polymeric matrix using the template molecule.

Additionally, Fe_3_O_4_-cored magnetic polymers were subjected to XRD analysis (Fig. 3b). The spectra present broadened edgeless bands between 10 and 20°, which strictly indicates the formation of the amorphous polymeric net on the surface of the nanoparticles. Based on the full width at half maximum (FWHM) values of the most intensive reflex, approximately at 35.4°, on each spectrum, the size of the magnetic polymers was estimated using Scherrer equation. The mean size of magNIP particles was 14.24 nm, while the imprinted materials TC–magMIP and DHMC-magMIP were 15.89 and 16.31 nm, respectively.

### 3.3. pH influence on the adsorption processes

**Fig. 4:**
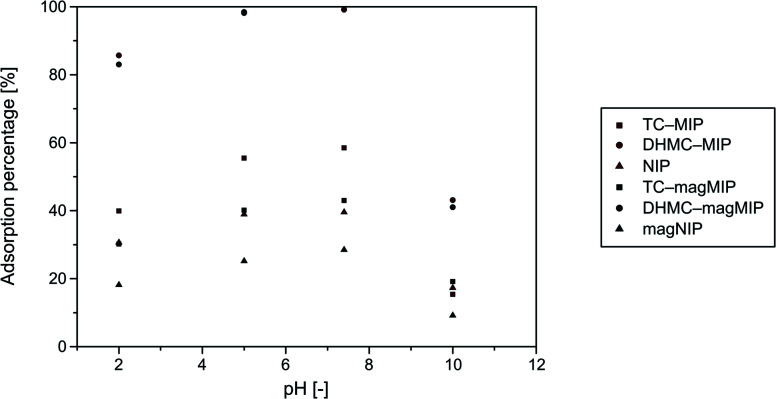
Dependence of 2′,4′-dihydroxy-3-methoxychalcone (DHMC) adsorption percentages on the synthesized polymers on the solutions pH values.

Sorptive properties of the synthesized polymers towards the used analytes are based on several factors, which are especially chemical structure of the chalcone enabling or hindering their binding within polymeric matrix on the basis of “lock-and-key” interactions, and also on the solutions' pH, since chalcone–polymer interactions are also achieved by the hydrogen bonding and acid–base stabilization. Therefore, an optimization of adsorption environment for all the materials studied and DHMC was performed. The choice of chalcone's dihydroxy-derivative as an exemplary analyte was driven mainly by the appearance of additional hydroxyl functional groups, which may undergo deprotonation under particular conditions, and thus highlight an influence of pH on the materials' sorptive potential. The adsorption experiments were handled in four different media, which were buffers of pH 2.0, 5.0, and 10.0, and phosphate-buffered saline (pH 7.4), which imitates physiological conditions. The dependence of adsorption percentages on the solutions pH is presented in Fig. 4.

The highest adsorption efficiencies were obtained for almost neutral conditions of pH 5.0 and 7.4. The adsorption of chalcone from the most acidic solutions of pH 2.0 was approximately 20% less effective, which was related to the excess of H^+^ ions disrupting hydrogen bonding and acid–base stabilization of polymer–chalcone complexes. On the other hand, the basic conditions (buffer of pH 10.0) led to a formation of anionic form of chalcone, which significantly hindered chalcone's binding to the acrylamide-based polymers by more than 50%. Therefore, the further adsorption studies were conducted in neutral systems of ethanol/water mixture.

### 3.4. Isothermal studies of acrylamide-based polymers

**Fig. 5:**
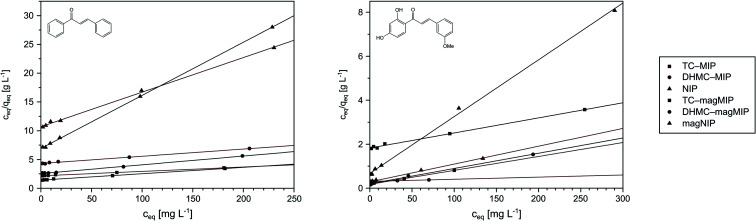
The Langmuir presentation of the isothermal studies of *trans*-chalcone (left) and 2′,4′-dihydroxy-3-methoxychalcone (right) adsorption on the obtained polymers.

Performing the isothermal studies is a key step for describing the adsorption processes, which leads to several adsorption parameters, helping in the understanding of the adsorbent-analyte system nature at equilibrium. Two of the most common adsorption isotherm models used are the Langmuir and the Freundlich models, which theories predominantly differ in the assumption of formation of adsorbate mono- or polylayer, respectively.^30^Table 2 presents the parameters and correlation coefficients of the experimental data fitting to the described isotherm models. In each adsorption process, the calculated *R*^2^ values are higher for the Langmuir model (>0.992), which indicates the better correlation of chalcones adsorption on MIPs and magMIPs to this model, graphically presented in Fig. 5. Accordingly, a very important parameter, which is maximal adsorption capacity *q*_m_, was calculated for all the adsorbents. The adsorption capacities of MIPs were found to be the highest towards the template used for their molecular imprinting, which proves the proper formation of specific recognition sites within the polymer net. The *q*_m_ values ascribed for the adsorption of TC were the highest for TC–MIP and its magnetic equivalent, reaching 131.58 and 90.33 mg g^−1^, respectively. *Trans*-chalcone adsorption on the materials imprinted with 2′,4′-dihydroxy-3-methoxychalcone was relatively less effective, which was mostly caused by the size differences between the cavities and the analyte, which hindered maximal saturation of the materials' empty binding sites. The non-imprinted materials showed no adsorption properties towards TC, which binding was based only on the analyte physical entrapment within the pores. The second chalcone studied was 2′,4′-dihydroxy-3-methoxychalcone, which adsorption was the most intense for DHMC–MIP and DHMC–magMIP, which *q*_m_ values reached 474.71 and 168.35 mg g^−1^, respectively, strictly proving the recognition of template by MIPs. The adsorption of DHMC might have been intensified by the presence of hydroxyl and methoxy pendant groups, able to non-covalently interact with the polymer net, which is visible for TC–MIP and TC–magMIP described with *q*_m_ values towards DHMC of ∼150 mg g^−1^, reaching higher values than those calculated for adsorption of their template molecule – *trans*-chalcone. Moreover, it is worthy to highlight that for all MIPs and NIPs their magnetic equivalents are less effective, which might be a reason of polymer matrix growth on Fe_3_O_4_/SiO_2_ nanoparticles, leading to a thin polymeric layer of decreased porosity and a slightly more difficult imprinting process. For the same polymer masses of MIPs and magMIPs, Fe_3_O_4_ core was a significant part of the magnetic polymers, therefore the number of binding cavities in magMIPs polymer layer is smaller.

**Table 2:** The isothermal data for chalcones adsorption on the synthesized MIPs and magMIPs

Polymer	Langmuir isotherm			Freundlich isotherm		
	*q*_m_ [mg g^−1^]	*K*_L_ 10^2^ [L mg^−1^]	*R* ^2^	1/*n* [−]	*K*_F_ [mg g^−1^ (L mg^−1^)^1/*n*^]	*R* ^2^
***Trans*-chalcone**						
TC–MIP	131.58 ± 4.64	0.35 ± 0.05	0.9975	0.91 ± 0.02	0.52 ± 0.04	0.9947
DHMC–MIP	79.68 ± 2.60	0.29 ± 0.02	0.9974	0.90 ± 0.02	0.26 ± 0.02	0.9938
NIP	27.54 ± 0.98	0.31 ± 0.03	0.9977	0.81 ± 0.04	0.11 ± 0.02	0.9896
TC–magMIP	90.33 ± 1.81	0.78 ± 0.09	0.9972	0.85 ± 0.04	0.47 ± 0.07	0.9902
DHMC–magMIP	65.62 ± 1.55	0.60 ± 0.05	0.9980	0.83 ± 0.04	0.85 ± 0.28	0.9912
magNIP	10.83 ± 0.08	1.33 ± 0.16	0.9997	0.72 ± 0.05	0.21 ± 0.05	0.9740

**2′,4′-Dihydroxy-3-methoxychalcone**						
TC–MIP	157.98 ± 4.79	3.61 ± 0.01	0.9996	0.71 ± 0.04	5.78 ± 0.03	0.9959
DHMC–MIP	474.71 ± 9.25	0.56 ± 0.03	0.9954	0.96 ± 0.01	3.31 ± 0.01	0.9841
NIP	93.30 ± 4.70	2.85 ± 0.23	0.9928	0.69 ± 0.04	4.05 ± 0.03	0.9834
TC–magMIP	145.77 ± 4.50	2.94 ± 0.38	0.9963	0.87 ± 0.03	0.67 ± 0.10	0.9703
DHMC–magMIP	168.35 ± 5.41	0.38 ± 0.08	0.9951	0.65 ± 0.05	5.55 ± 0.04	0.9948
magNIP	38.71 ± 0.84	3.84 ± 0.16	0.9976	0.52 ± 0.06	2.37 ± 0.10	0.9354

**Fig. 6:**
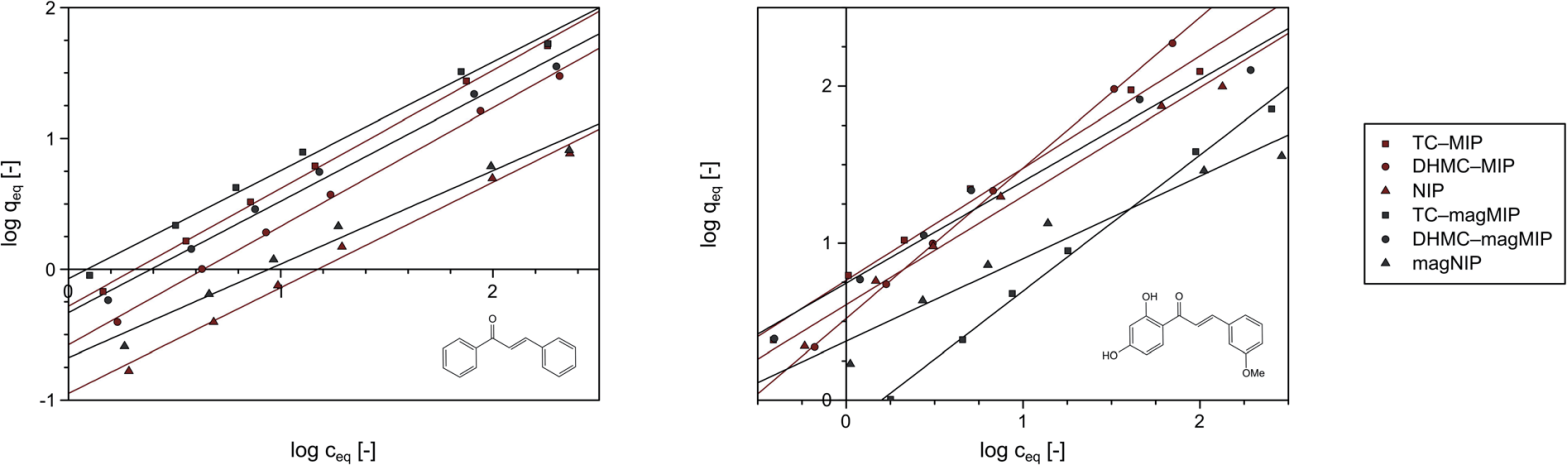
The fitting of the experimental data to the Freundlich adsorption model for *trans*-chalcone (left) and 2′,4′-dihydroxy-3-methoxychalcone (right).

Nevertheless, the fitting of the experimental data to the Freundlich model (Fig. 6) gave reliable correlation coefficients *R*^2^, which values varied between 0.9354 and 0.9948. Therefore, values of the empirical 1/*n* parameter were calculated for each of the adsorption processes, which is connected with the process intensity (Table 2). Namely, when the parameter value is between 0 and 1, the adsorption process is favorable and is more intense with the value increase. For the chalcones adsorption on MIPs and NIPs, 1/*n* values ranged between 0.72 and 0.91 for *trans*-chalcone adsorption, and between 0.52 and 0.96 for 2′,4′-dihydroxy-3-methoxychalcone adsorption, indicating high affinity of the materials towards the used analytes.

The obtained polymer-template complexes were also further subjected to the chalcones' release in various aqueous conditions, which were buffer solutions of pH 2.0, 5.0, 7.4, and 10.0. Although the desorption processes were mostly described as inefficient after 2 hour release period, the visible pH influence was observed for DHMC release from the polymer matrices in basic environment. An influence of solution's environment is significant only if chalcone contains pendant pH-labile functional groups, such as hydroxyl, amine, or carboxyl. The studied *trans*-chalcone has no additional groups attached to none of the aromatic rings, therefore formation and/or deformation of polymer–TC complex is based only on pH-independent complementary supramolecular recognition. On the other hand, the studied dihydroxy-derivative of chalcone may undergo deprotonation under basic conditions, leading to mono- or dianionic form, triggering repulsive interactions with basic domains of polymeric net – acrylamide. Therefore, a significant release of bound DHMC was observed only after polymer–DHMC complex incubation in buffer of pH 10.0, which led to 30% chalcone's release.

### 3.5. Investigation of adsorption of various chalcones

According to the findings described in paragraph 3.3, proving the binding ability of the obtained polymers not only towards the template molecule, studies aiming at the investigation of four structurally different chalcones adsorption – *trans*-chalcone (TC), 2′,4′-dihydroxy-3-methoxychalcone (DHMC), 4-hydroxychalcone (HC), and 4-methoxychalcone (MC) – were conducted.

#### 3.5.1. Influence of the materials dose

The polymer samples of 10, 20, and 30 mg were incubated with a mixture of the chalcones at their final concentration of 0.1 mM. Therefore, an influence of the materials' doses on the binding efficiency might be easily described. The adsorption progress was monitored with ESI-MS analysis, which gave the analytical signals of each of the chalcone in positive mode (*m*/*z* 209 for TC and *m*/*z* 239 for MC) and negative mode (*m*/*z* 223 for HC and *m*/*z* 269 for DHMC). Basing on the signals' intensities in the spectra of solutes injected after the incubation with polymers, and the pre-performed dependence of the signal intensity and the chalcones concentration, the percentages of the chalcones adsorption were calculated (Table 3). The adsorption of template molecules by imprinted polymers was very high, even when the minimal amount of the polymers was used, and rose with the materials dose increase (TC entry for TC–MIP and TC–magMIP; DHMC entry for DHMC–MIP and DHMC–magMIP). For the highest MIPs doses, the adsorption percentages of the template molecules varied between 97.20 and 99.99%. Interestingly, the materials imprinted with *trans*-chalcone exhibits higher affinity for binding HC than other non-template chalcones, which might be a result of its smallest substituent hindrance responding to the size of TC cavities, reaching adsorption percentage values between 74.15 and 90.66% for TC–MIP, and 52.07 and 76.51% for TC–magMIP. Nevertheless, more sterically expanded DHMC and MC also show moderate affinity towards these materials, taking diffusion into materials' pores and moderate affinity towards TC cavities into account. On the other hand, the polymer imprinted with 2′,4′-dihydroxy-3-methoxychalcone and its magnetic equivalent exhibit satisfactory adsorptive properties towards MC, which is the most sterically hindered non-template analyte, which adsorption percentage reached even 93.87 and 91.65% for the highest dose of DHMC–MIP and DHMC–magMIP, respectively. Such results show that after the full saturation of MIPs cavities selective towards the template molecules, the polymers are able to bind other template-like molecules, starting with the most similarly substituted molecules. The binding process might involve the further saturation of cavities within the polymeric matrix, as well as diffusion into the material's pores or non-covalent interaction with the polymer's constituents, which is proved by moderate adsorption of the studied chalcones by non-imprinted polymers.

Adsorption percentages of each chalcone after MIPs, magMIPs, and NIPs incubation in the chalcones mixture

**Table:** 

Chalcone		TC–MIP			DHMC–MIP			NIP		
		10 mg	20 mg	30 mg	10 mg	20 mg	30 mg	10 mg	20 mg	30 mg
% adsorbed	TC	95.41	97.75	98.67	38.25	42.66	49.72	68.81	84.19	88.09
	DHMC	59.19	60.90	60.95	99.98	99.99	99.99	67.77	76.26	79.25
	HC	74.15	81.27	90.66	78.29	81.85	89.96	26.25	31.49	48.16
	MC	58.74	66.57	79.88	82.04	91.47	93.87	39.53	44.74	45.93

**Table:** 

Chalcone		TC–magMIP			DHMC–magMIP			magNIP		
		10 mg	20 mg	30 mg	10 mg	20 mg	30 mg	10 mg	20 mg	30 mg
% adsorbed	TC	82.82	88.64	97.20	45.73	47.08	52.38	45.52	47.46	53.48
	DHMC	43.48	55.83	60.16	99.18	99.83	99.96	28.90	37.95	43.31
	HC	52.07	61.67	76.51	71.12	75.37	85.13	29.60	35.51	52.71
	MC	56.75	60.66	62.87	87.70	90.04	91.65	14.26	20.16	34.81

#### 3.5.2. Kinetics of chalcones adsorption

**Fig. 7:**
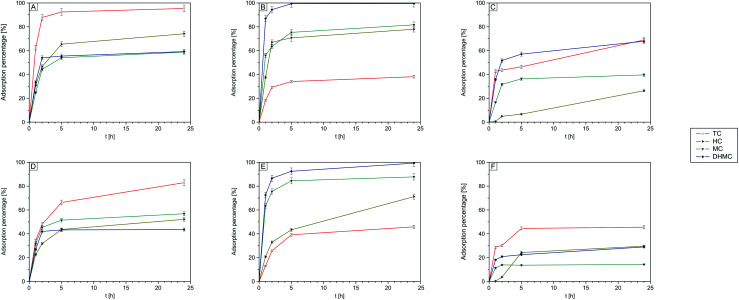
Kinetics of adsorption of chalcones on the synthesized polymers: TC–MIP (A); DHMC–MIP (B); NIP (C); TC–magMIP (D); DHMC–magMIP (E); magNIP (F).

The adsorption profiles deliver key information on the time needed for saturation of the adsorbent's binding sites with a particular analyte, as well as on the adsorption order from multi-ingredient solutions. The kinetics of adsorption of four structurally different chalcones from their mixture on the obtained imprinted and non-imprinted polymers was monitored using ESI-MS analysis, as described in paragraph 3.4.1. The adsorption profiles presented in Fig. 7 show the potential of MIPs (Fig. 7A and B), and NIP (Fig. 7C) and their magnetic equivalents (Fig. 7D–F) to adsorb the chosen chalcones. The adsorption of 2′,4′-dihydroxy-3-methoxychalcone on DHMC–MIP (Fig. 7B) and DHMC–magMIP (Fig. 7E) is very efficient, reaching adsorption percentage at 94.8 and 86.5%, respectively, after 2 hours. The full adsorption of more than 99% of analyte takes place after 5 hours for non-magnetic polymer, and after 24 hours for Fe_3_O_4_-based material. As it was before-postulated, the DHMC-imprinted materials show satisfactory adsorptive potential also towards MC, binding more than 70% already after 2 and 5 hours for DHMC–magMIP and DHMC–MIP, respectively. On the other hand, the materials imprinted with *trans*-chalcone (Fig. 7A and D) exhibit good selectivity towards template molecule. The significant saturation with template molecules is visible already after 2 hours for TC–MIP and 5 hours for TC–magMIP. The non-imprinted polymers, which adsorption profiles are presented in Fig. 7C and F, are capable of binding all the chalcones in no specific manner, as a consequence of their diffusion into polymers' non-selective pores on the surface. Nevertheless, NIP with magnetite core exhibits undoubtedly lower adsorptive potential towards the analytes, which may be attributed to the formation of the polymeric shell on Fe_3_O_4_ nanoparticles, slightly hindering elution to the materials' pores.

#### 3.5.3. FAPA-MS analysis of polymer–chalcone complexes

**Fig. 8:**
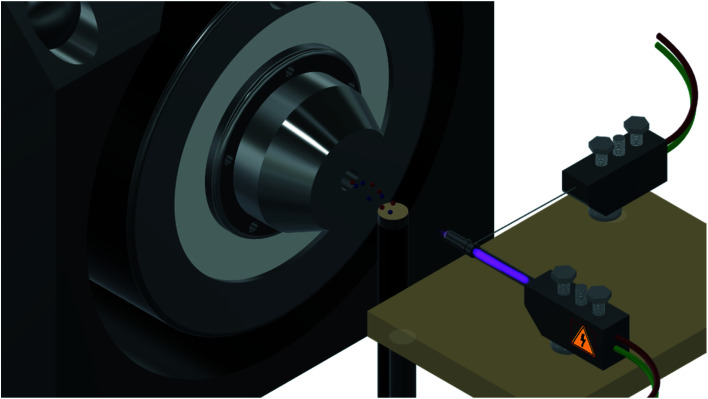
Schematic representation of MS analysis using flowing atmosphere-pressure afterglow (FAPA) ionization mode: the plasma is generated from an inert gas flowing between two electrodes (right); the sample placed on the crucible is introduced to the plasma in order to ionize all the analytes (center); the analytes are directed to the spectrometer (left).

Successful adsorption of the chosen chalcones on each of the polymeric materials was also investigated using the mass spectrometer equipped with flowing atmospheric-pressure afterglow (FAPA) ionization source. The method has already been successfully implemented for analysis of *e.g.*, non-steroidal anti-inflammatory agents, volatile organic compounds, hormones, flavonoids, pesticides, or antioxidants, and is a very suitable technique for the fast and reliable detection of a wide range of analytes, omitting a demanding pre-separation or extraction steps.^26,31–36^ The main feature of FAPA ionization mode is connected with an external ionization source, which is a plasma generated from helium flowing between two electrodes. Analytes introduced to the plasma are immediately ionized and directed to the spectrometer through the flowing gas (Fig. 8). In the case of analytes, which ionization is possible only at elevated temperature, the sample crucible can be heated up to 300 °C, with a remained temperature control, allowing the full ionization. Such an approach is facilitating the sample introduction to the ionization source by the possibility of analysis of pure solid analytes, aqueous or non-aqueous solutions, analytes bound to adsorptive surface (silica gel, filter paper, or fabrics), host–guest complexes, and other non-covalently bound analytes.

**Fig. 9:**
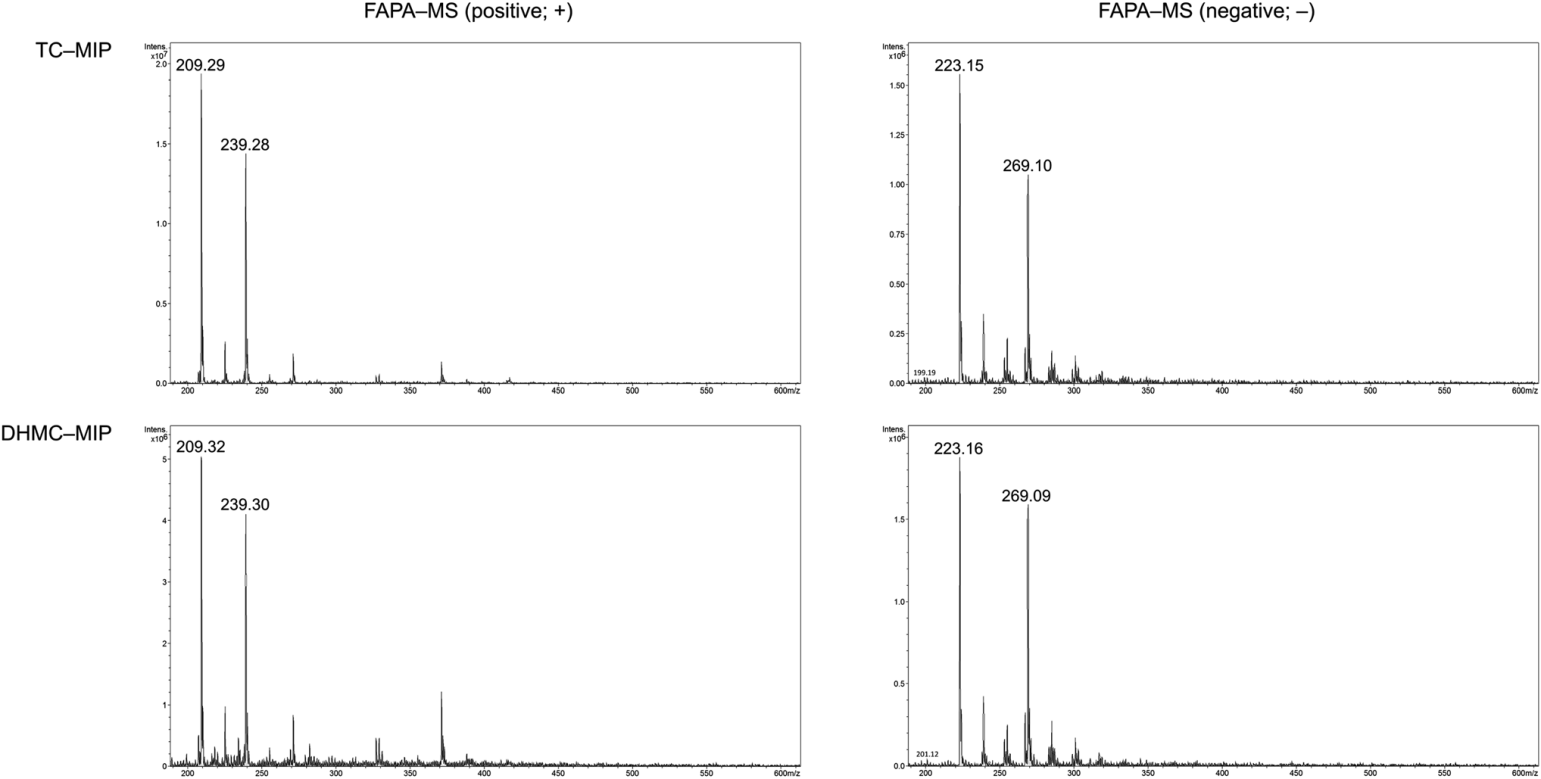
The FAPA-MS spectra of MIPs complexed with the studied chalcones, performed in positive and negative modes.

**Fig. 10:**
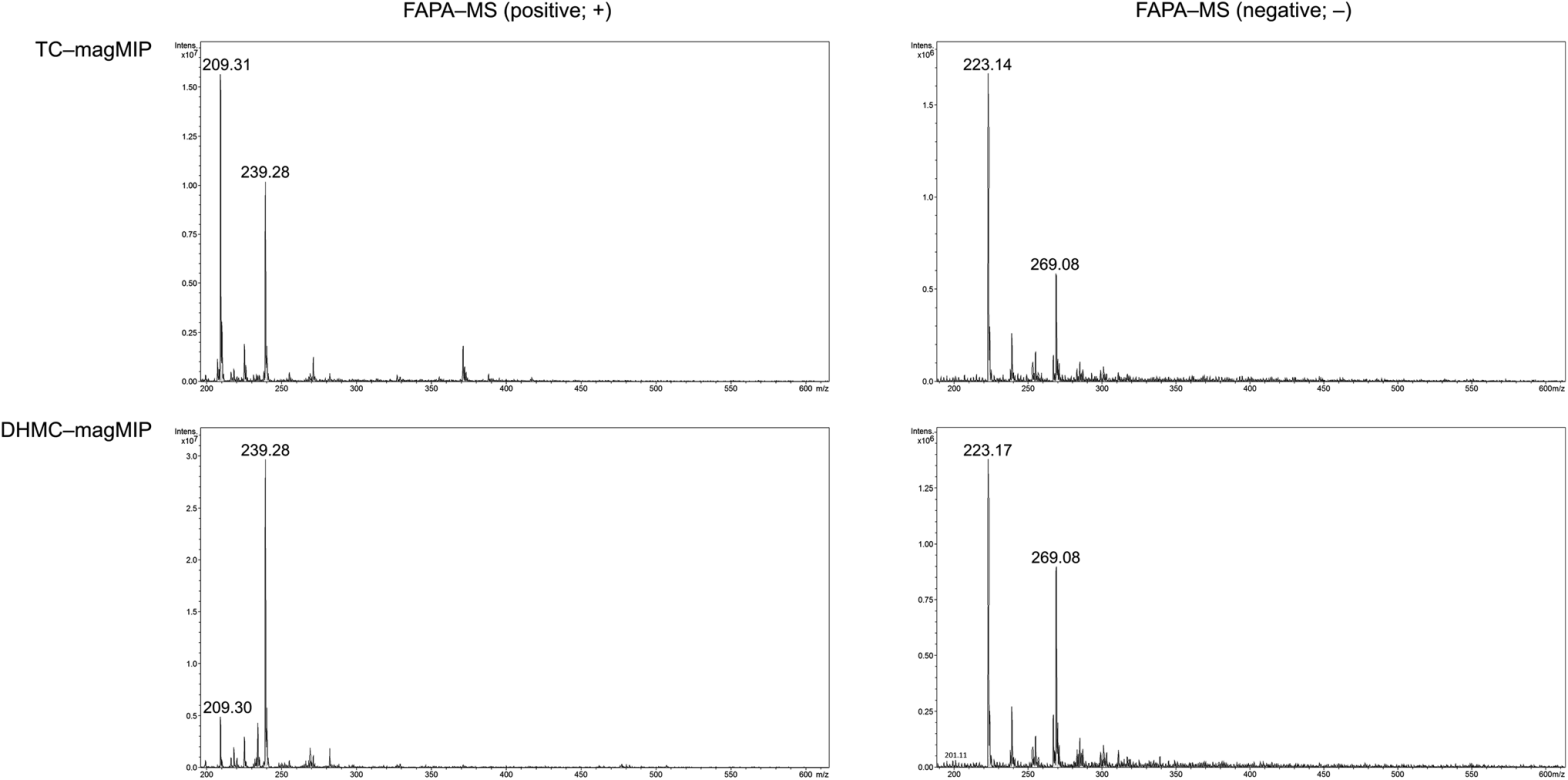
The FAPA-MS spectra of magMIPs complexed with the studied chalcones, performed in positive and negative modes.

The adsorption of the chalcones within the polymeric matrices is based on non-covalent host–guest interactions, which are crucial for the application of FAPA-MS technique. The spectra of the complexes of the synthesized polymers and their magnetic equivalents with four chalcones (TC, HC, MC, and DHMC) used for kinetic measurements are presented in Fig. 9 and 10, respectively.

For both the template molecules, *trans*-chalcone and 2′,4′-dihydroxy-3-methoxychalcone, the signals corresponding to their molecular peaks at *m*/*z* 209 in positive mode and *m*/*z* 269 in negative mode, respectively, are the highest for the materials imprinted with these chalcones. It is worthy to highlight that the recorded FAPA-MS spectra presented the same signal profiles as the ones recorded using ESI-MS technique. This is consistent with previously conducted studies on MS^n^ analysis of various chalcones recorded using both mentioned techniques, which showed no differences in the samples spectra triggered by the use of particular ionization mode.^37^ The intensities reached even 1.95 × 10^7^ and 1.55 × 10^7^ for TC–MIP and TC–magMIP, respectively, and 1.6 × 10^6^ and 0.9 × 10^6^ for DHMC–MIP and DHMC–magMIP, respectively. The differences between the order of magnitudes of these values are caused by the spectrometer working modes for the detection of particular signals. *Trans*-chalcone signal intensities at *m*/*z* 209 decrease in the order TC–MIP > NIP > DHMC–MIP, while for 2′,4′-dihydroxy-3-methoxychalcone, this order is given as DHMC–MIP > TC–MIP ≫ NIP, basic on the intensities of signal *m*/*z* 269. Therefore, it can be assumed that the most compact chalcone (TC) diffuse and bind more easily to NIPs pores, based on the physical entrapment, than to non-complementary cavities of DHMC-imprinted polymers. Expect of *trans*-chalcone, NIP and magNIP bind the substituted chalcones with the least efficiency, indicating their better suitability to the cavities of the imprinted polymers than structural pores formed during the materials' syntheses. All the results are consistent with the conclusions drawn for the kinetic studies described in paragraph 3.4.2. Moreover, the possibility of the thermal desorption of chalcones proved by the performed FAPA-MS analyses, may indicate a possible application of the obtained MIPs as pre-concentrating tools in quantitative and/or qualitative analyses, using several analytical techniques including mass spectrometry, thermogravimetric measurements, or chromatographic analysis.

## 4. Conclusions

The current study showed a method for obtaining the chalcone-imprinted polymers with their further application as adsorbents towards various chalcone molecules. The polymers' synthesis was optimized by a choice of the most suitable functional monomer, affording the most efficient creation of the chalcone-complementary cavities within the polymeric matrices. The imprinted and non-imprinted polymers were obtained *via* direct polymerization in the reaction mixtures or immobilized on the surface of the magnetite nanoparticles. All the materials were tested for adsorption of template molecules, as well as structurally different chalcones as guest molecules. The interactions between the materials and analytes were investigated by isothermal and kinetic studies, indicating very efficient adsorptive properties of the obtained polymers. Although the materials showed satisfactory adsorption selectivity towards template molecules, the binding of structurally different chalcones was also found to be intensive. Moreover, Fe_3_O_4_-based materials showed proper magnetic susceptibility, which facilitated their analytical application. Concluding, the use of different analytical techniques in chalcones adsorption investigation, including UV-vis measurements and MS analysis performed using two different ionization sources, undoubtedly proved the sorptive effectiveness of the obtained MIPs towards the chosen chalcones. Therefore, the proposed materials may be also applied as useful tools for detection, pre-concentration, or transport and distribution of variously bioactive chalcones.

## Conflicts of interest

The authors declare no known financial or personal conflicts of interest associated with this publication.

## Supplementary Material
